# Characterization of the Differences in Dissolved Organic Matter (DOM) Adsorbed on Five Kinds of Microplastics Using Multiple Methods

**DOI:** 10.3390/molecules30071586

**Published:** 2025-04-02

**Authors:** Xianshu Fu, Xiangliang Pan, Jun Chen, Mingzhou Zhang, Zihong Ye, Xiaoping Yu

**Affiliations:** 1 College of Environment, Zhejiang University of Technology, Hangzhou 310032, China; fxs@cjlu.edu.cn; 2 Key Laboratory of Microbiological Metrology, Measurement & Bio-Product Quality Security, State Administration for Market Regulation, College of Life Sciences, China Jiliang University, Hangzhou 310018, China; zmzcjlu@cjlu.edu.cn (M.Z.); zhye@cjlu.edu.cn (Z.Y.); yxp@cjlu.edu.cn (X.Y.); 3 College of Biological and Environmental Engineering, Zhejiang Shuren University, Hangzhou 310021, China; 4 Laboratory of Pollution Exposure and Health Intervention Technology, Interdisciplinary Research Academy, Zhejiang Shuren University, Hangzhou 310021, China

**Keywords:** microplastics (MPs), dissolved organic matter (DOM), total organic carbon (TOC), gel permeation chromatography (GPC), excitation-emission matrix-parallel factor analysis (EEM-PARAFAC)

## Abstract

Microplastics (MPs) are ubiquitous in aquatic environments, soils, and beach sediments, demonstrating a remarkable ability to adsorb dissolved organic matter (DOM). Although there are methods for extracting DOM from water, the approaches for directly extracting DOM from microplastics have not been thoroughly investigated, and the characterization of DOM adsorbed on microplastics is also insufficient. In this study, five different types of microplastic samples were collected from each of five environmental media (water and sediment), and finally 25 samples were obtained. This paper comparatively assessed the extraction efficiency of DOM from MPs with various solvents by using total organic carbon (TOC), culminating in the development of a sodium pyrophosphate-NaOH solution extraction method optimized for DOM. The morphology, material and environmental medium of microplastics were the three primary factors affecting the adsorption of DOM on microplastics, with the highest enrichment ratio of 1.4–1.8 times for extruded polyethylene microplastics (EPE-MPs) characterized by their porous structure in the flowing water environment. The molecular weight of DOM adsorbed on microplastics showed a multi-modal distribution pattern with great dissimilarities among the different environmental media. Gel permeation chromatography (GPC) indicated that the weight-average molecular weight (*M_w_*) of DOM was 2750–4552 Da for river MPs, 2760–5402 Da for Qiantang River MPs, 1233–5228 Da for East China Sea MPs, 440–7302 Da for soil sediment MPs and 438–6178 Da for beach sediment MPs, respectively. Excitation-emission matrix-parallel factor analysis (EEM-PARAFAC) identified that tyrosine-like substances with high excitation in region IV and low excitation in region I were predominantly adsorbed on MPs, followed by tryptophan-like substances with low excitation in region II and protein-like substances in region IV, while humic- and fulvic-like substances in regions V and III, respectively, exhibited the least adsorption affinity. The findings underscored the critical need to comprehensively consider the interactions between MPs and DOM and their environmental impacts in pollution control strategies.

## 1. Introduction

Microplastics (MPs), defined as plastic particles smaller than 5 mm [1], have emerged as the number one scientific issue in the field of environmental and ecological sciences since their introduction by Thompson et al. in 2004 [2,3]. These particles, ranging from millimeters to nanometers in size, are pervasive in terrestrial, marine and atmospheric pollution sources, with over 80% of marine MPs originating from terrestrial sources [4,5,6,7,8]. MPs are predominantly transported into oceans via rivers, stormwater drainage systems, and sewage outflows [9,10], contributing an estimated 1.15–2.41 million tons of plastic waste annually [11]. In terrestrial ecosystems, potential pathways for MPs introduction include the use of ground plastic films, the reuse of treated wastewater effluent, irrigation practices utilizing domestic or industrial wastewater, and runoff derived from tire wear and improperly disposed household plastic waste [12,13,14].

Due to their small size and larger specific surface area, MPs can adsorb environmental pollutants such as pathogens [15], antibiotics [16], and a spectrum of organic/inorganic contaminants [17,18], acting as carriers or substrates for these substances—a phenomenon known as the “vector effect” [19,20]. This interaction facilitates the transfer of harmful chemicals to marine organisms, potentially inducing widespread biological toxicity. MPs could also trigger oxidative stress and cellular damage in organisms through physical interactions such as abrasion [21]. Additionally, MPs smaller than 1 µm pose direct human health risks by penetrating the circulatory system and inducing cellular and molecular damage [22,23,24].

MPs exhibit a strong affinity for dissolved organic matter (DOM) [25,26]. DOM, defined as organic matter soluble in acid, alkali solutions, or water, is one of the most complex [27], heterogeneous, and diverse mixtures known, with only a fraction of its components structurally characterized. The representative structures and active parts of DOM include functional groups such as alkenes, quinones, amines, phenols, and heterocyclic groups [28], with corresponding macromolecules containing carbohydrates, polysaccharides, amino acids, peptides, proteins, lipids, humic substances, and anthropogenic organic pollutants [29,30]. The concentration and composition of DOM vary significantly across environments, depending on sources and transformation processes [31,32]. Hydrological conditions, precipitation, and human activities are pivotal parameters influencing the quantity and composition of DOM in the natural waters, drinking water sources, and lake ecosystems. Humic substance components predominantly occur in upstream tributaries, while protein components are more commonly found in lake environments. Water enriched with allochthonous DOM tends to be more hydrophobic, with higher aromatic content and greater ultraviolet-visible light absorption [33].

Studies have demonstrated that the adsorption of DOM onto MPs is influenced by both microplastic properties and environmental conditions. For instance, polystyrene (PS) microplastics in aquatic systems can absorb DOM components such as humic acid (HA), fulvic acid (FA) and tannic acid (TA), with these processes often aligning with the Freundlich isotherm model and pseudo-second-order kinetic model. Adsorption capacity varies with environmental factors such as pH changes [34,35]. For instance, HA and FA adsorption is more pronounced under acidic conditions, while TA adsorption peaks at specific pH values. DOM adsorption not only alters the environmental behavior of MPs but also results in the release of these substances through mechanical, photochemical, and biodegradation processes, thereby impacting ecosystems [36,37]. Research indicates that the presence of DOM can modify the surface properties of MPs, affecting their capacity to adsorb other pollutants.

Common methodologies for DOM separation include physical or chemical precipitation, liquid-liquid extraction (LLE), solid-phase extraction (SPE), XAD macroporous resin adsorption, membrane separation, and ultrafiltration techniques. The XAD resin technique, endorsed by the International Humic Substances Society, is the standard method for extracting humic substances. However, these conventional methods are often inadequate for extracting DOM adsorbed onto microplastic particles and face challenges such as low extraction yields and an inability to fractionate based on molecular weight [38]. For soluble organic matter in matrices like rocks and crude oil, standards such as SY/T 5118-2021 [39] and SY/T 5119-2016 [40] utilize dichloromethane and trichloromethane as extraction solvents, followed by purification using organic solvents of varying polarities to isolate the target DOM. However, these organic solvents may interact with MPs through intermolecular forces or chemical reactions, leading to melting, deformation, degradation, alterations in molecular chains, or introduction of new functional groups. Additionally, some organic solvents can physically adsorb onto microplastics, filling intermolecular gaps and modifying their strength, hardness, and other physical properties.

There has been some exploration in the extraction methods for DOM and humic substances in different minerals. Abroad, purification and distillation technologies are employed to refine humic acid to different levels according to customer specifications, with instruments such as UV spectrophotometers and spectrometers used for determination, yielding humic acids of varying molecular weights [41,42]. In China, sodium pyrophosphate solutions, sodium pyrophosphate alkaline solutions, or sodium hydroxide alkaline solutions are commonly utilized to extract humic acid, total humic acid, and free humic acid from lignite, respectively. Li et al. [43] extracted humic acid from typical Yunnan lignite using sodium pyrophosphate alkaline solution and sodium hydroxide, finding that the sodium pyrophosphate alkaline solution contained a higher proportion of low molecular weight humic acid with higher ash content.

However, direct research on the extraction of DOM from MPs in various environmental media remains limited. In environmental microplastic pollution, polyethylene (PE) and polystyrene (PS) microplastics are the most prevalent, followed by polypropylene (PP) and polyethylene terephthalate (PET) microplastics [44]. Therefore, it is essential to focus on the most common microplastic pollutants in different environments, analyze the DOM adsorbed on them, and conduct differentiated studies of the extracted DOM.

## 2. Materials and Methods

### 2.1. Microplastic Samples

**Figure 1:**
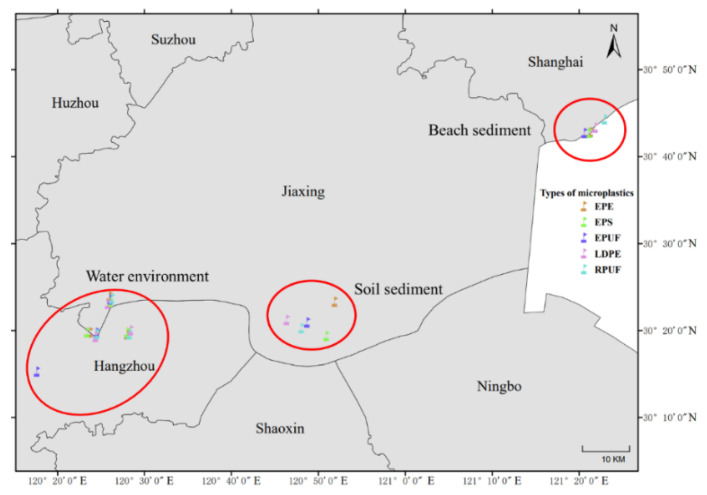
Geographical map of microplastic sampling site.

Hangzhou, Haining, and Shanghai were selected as the strategic sampling sites for the collection of microplastics. The study encompassed a range of materials, including expandable polyethylene (EPE), expanded polystyrene (EPS), flexible polyurethane foam (FPUF), rigid polyurethane foam (RPUF) and low-density polyethylene (LDPE), which were retrieved from inland river waters (River), the Qiantang River (QTR), the East China Sea (ECS), soil sediments (SS), and beach sediments (BS). To mitigate the variability in DOM content attributable to disparate environmental media, the sampling sites for the five microplastic types were carefully chosen in close proximity, with no more than 3000 m separating any two sampling points. Concurrently, water and sediment samples from the same locations as the microplastic collections served as control groups. A total of 25 microplastic samples and 25 corresponding control samples were obtained, the details of which are shown in Table 1 and Figure 1.

### 2.2. Reagents and Equipment

Dextran series standards with molecular weights of 180, 342, 505, 1300, 2800, 5000, 12,000, 25,000, 49,600, and 62,900 Da (500 mg/bottle, ultra-pure) were purchased from Sigma-Aldrich Company (Burlington, MA, USA). Na_2_HPO_4_ (25 g/bottle, chromatographic purity), NaH_2_PO_4_ (100 g/bottle, chromatographic purity) and NaCl (25 g/bottle, chromatographic purity) were from Sinopharm Group Chemical Reagent Co., Ltd. (Shanghai, China). Sodium pyrophosphate (100 g/bottle, analytical purity) and sodium hydroxide (500 g/bottle, analytical purity) were acquired from Shanghai Macklin Biochemical Technology Co., Ltd. (Shanghai, China) and Zhejiang Hanno Chemical Industry (Jinhua, China), respectively. Ultrapure water was produced from ultrapure water meter (Direct-Q 5UV, Merck Millipore, Darmstadt, Germany). The separation was performed using a benchtop high-speed refrigerated centrifuge (Velocity 18R Pro, Dynamica, Shenzhen, China). DOM characteristics were characterized by TOC analyzer (TOC-L, Shimadzu, Kyoto, Japan), gel permeation chromatography (GPC) (Arc HPLC, Waters, Milford, MA, USA), and excitation-emission matrix (EEM) fluorescence spectroscopy (F-4700, Hitachi, Tokyo, Japan). All glassware was washed three times with ultrapure water before the experiment, dried, and then set aside for use.

### 2.3. Extraction Methods of DOM Adsorbed on Microplastics in Different Environmental Media

The strongly alkaline NaOH does have a certain dissolution effect on the surface of microplastics, but this effect varies according to the material and treatment conditions of microplastics. The study of Dehaut et al. [45] showed that 10 M NaOH, after 60 °C and 24 h, would not destroy the structure of most microplastics such as PE, PS, PUR, PP, PMMA, and only wreck the structure of CA, PC, PET, and PVC. The above research indicates that high concentrations of NaOH are corrosive to only a few specific microplastics at high temperatures and for a long time, and can change the physical properties of microplastics, especially the stability of their internal additives. Hurley et al. [46] also found similar behavior described above at 60 °C, 1 M NaOH, and 24 h. Cao et al. [47] mentioned that in a high-temperature alkaline solution, NaOH can hydrolyze the dioctyl phthalate (DOP) plasticizer in flexible PVC, which further demonstrates the ability of NaOH to dissolve additives in specific kinds of microplastics.

This paper delved into the methodologies for extracting DOM from disparate environmental matrices, utilizing ultrapure water (UPW) and sodium pyrophosphate-NaOH solution as the DOM extraction reagents. The study encompassed the extraction of DOM from aquatic environments, soil and beach sediments, as well as from soil and beach themselves. The extracted solutions were subsequently analyzed for total organic carbon (TOC), inorganic carbon (IC) and total carbon (TC) employing TOC analyzer. The comparative analysis of extraction efficiency for DOM by different reagents was conducted based on the content of TOC and IC, thereby ascertaining the most optimal extraction scheme. First, two aliquots of 0.5 g from the same type of microplastic fragments were, respectively, extracted with 20 mL ultrapure water and 20 mL sodium pyrophosphate-NaOH solution (comprising 0.03 mol/L sodium pyrophosphate and 0.01 mol/L sodium hydroxide) for 10 min at room temperature and under ultrasonic condition. Subsequently, the extraction solution was centrifuged at 10,000 rpm to obtain the extraction liquid of ultrapure water (labeled UPW Group) and sodium pyrophosphate (labeled Pyrophosphate Group), respectively. The microplastic fragments extracted with ultrapure water were then drawn out with 20 mL sodium pyrophosphate-NaOH solution to obtain a secondary extraction solution (designated as UPW then Pyrophosphate Group). Each microplastic type was processed in triplicate using the above method, and the concentrations of the UPW Group, Pyrophosphate Group, and UPW then Pyrophosphate Group were quantified, with the mean values being taken for analysis. By comparing the levels of TOC and IC, the most effective extraction technique was ultimately identified.

For the extraction of DOM inherent in soil and beach sediments, the remaining conditions were consistent with the above method except that the extractant and MP samples were 10 g and 100 mL, respectively. Finally, three groups of extracts were acquired.

### 2.4. Scanning Electron Microscopy (SEM)

The surface morphologies of five different types of microplastics were identified by scanning electron microscopy (SEM, ZEISS GeminiSEM 360, Oberkochen, Germany) before and after extraction. The microplastic samples were directly glued to the sample table with conductive adhesive, then gold was sprayed, and the working voltage was 3 kV by SEM.

### 2.5. Detection of TOC Content in DOM

Using the optimal extraction method identified above, DOM was achieved from microplastics collected from River, QTR, ECS, SS, and BS as listed in Table 1, and the content of TOC, among other things, was determined. TOC serves as a comprehensive indicator that indirectly reflects the organic matter content in water bodies, representing the total carbon content of organic substances present, expressed in units of carbon (c) mg/L. Typically, the TOC levels in urban sewage can reach up to 200 mg/L, while industrial wastewater exhibits a broader range, with the highest values reaching tens of thousands of mg/L. Post-secondary biological treatment, sewage generally exhibits TOC levels below 50 mg/L. Detection of TOC and TC is conducted using a Total Organic Carbon Analyzer, adhering to the differential method outlined in the HJ 501-2009 standard [48], “Determination of Total Organic Carbon in Water Quality by Combustion Oxidation-Non-Dispersive Infrared Absorption Method”.

Parameters of the TOC Analyzer include the use of a fixed temperature catalytic combustion oxidation method at 680 °C; a high-sensitivity Non-Dispersive Infrared (NDIR) detector combined with a large sample volume combustion system, which theoretically can detect TOC below 10 µg/L (0.01 mg/L) and IC below 4 µg/L (0.004 mg/L). However, the practical and reliable detection range of the analyzer is 0.5 mg/L. While detection below this threshold is technically possible, it is not recommended due to significant noise interference, weak signal strength, and poor data feasibility, which can adversely affect the accuracy of the detection results.

### 2.6. Molecular Weight Distribution of DOM

The GPC analyzer utilized in this study is outfitted with the following parameters: it is equipped with an Acquity UPLC Protein BEH SEC Column, which has specifications of 125 A, 1.7 μm particle size, and dimensions of 4.6 mm × 300 mm, with a pack size of 1/pk. The system is configured with a Photodiode Array (PDA) detector, calibrated to a detection wavelength of 254 nm. The column temperature is maintained at 30 °C, and the flow rate is set to 0.3 mL/min. The injection volume for samples is 10 μL, with the cell temperature also controlled at 30 °C. The mobile phase consists of an aqueous solution containing 0.001 M Na_2_HPO_4_, 0.001 M NaH_2_PO_4_, and 0.03 M NaCl. For calibration, a series of dextran standards ranging from 180 to 62,900 Da are employed to generate a calibration curve, ensuring the accuracy and reliability of the molecular weight measurements.

### 2.7. Analysis of DOM Fluorescence Characteristics

Excitation-emission matrix (EEM) fluorescence spectrum, the measurement parameters are as follows: (1) excitation light source, 150 W xenon lamp, (2) 700 V PMT voltage, (3) SNR > 110, (4) full band scanning, (5) excitation spectrum (Ex = 200~730 nm) and emission spectrum (Em = 200~730 nm), (6) 5 nm step length, (7) 2400 nm/min scanning speed, (8) 1 cm special quartz colorimetric dish, (9) 1 nm resolution, (10) FL solutions 2.4 software, and (11) MATLAB R2022b (9.13.0.2049777). To remove Raman scattering, the fluorescence spectra of ultrapure water were taken as blank. And the EEM of ultrapure water is used to monitor the stability of the fluorescence spectrophotometer. If there is no instrument error, if not, the F-4700 fluorescence spectrophotometer (Hitachi, Tokyo, Japan) needs to be recalibrated. Before the test, the sample should pass through a 0.45 µm filter membrane, and the sample concentration should not be higher than 10 mg/g, because high DOC content is prone to fluorescence quenching.

## 3. Experimental Results and Discussion

### 3.1. Extraction Procedure of DOM Adsorbed on Microplastics

To investigate the influence of different environmental media on the extraction of DOM, the microplastics collected from water environment, soil and beach sediments were extracted to obtain the DOM adsorbed on microplastics. At the same time, the DOM of the environmental medium in the microplastics collection site was used as the control group.

#### 3.1.1. Extraction Approach of DOM Adsorbed on Microplastics from River

The DOM in River-1^a^ microplastics and the DOM inherently present in the river water samples (control group) were extracted and separated using the aforementioned methods, and the TOC content was determined. The results are presented in Table 2.

As shown in Table 2, the impact of treatment methods in DOM release is significant. Pyrophosphate-treated MPs exhibited the highest TOC concentrations across all MP types (e.g., 4.37 mg/L for EPE-MPs), significantly surpassing the control group (River-1: 2.39–3.07 mg/L). This indicates that pyrophosphate, likely through chelation or surface disruption, effectively mobilizes organic carbon bound to MPs. Notably, the IC values also increased proportionally (e.g., 23.03 mg/L IC for EPE-MPs), suggesting that the treatment liberates both organic and inorganic fractions. In contrast, UPW treatment resulted in minimal TOC release (e.g., 1.00–1.73 mg/L for LDPE-MPs and EPE-MPs), which was even lower than natural river water. This implies that DOM release from MPs cannot account for all the DOM in natural environments under passive leaching. The TOC levels in the combined treatment (UPW then Pyrophosphate) group (e.g., 1.04–2.85 mg/L) were higher than UPW alone, manifesting that the TOC in microplastics could not be completely extracted with ultrapure water as the extraction agent.

**Figure 2:**
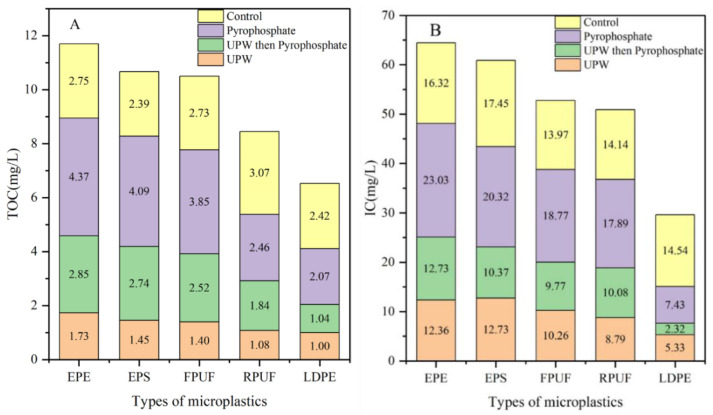
Contents of DOM in River-1^a^ under different pretreatment procedures, where (**A**) and (**B**) were TOC and IC concentrations, respectively.

Using Origin software (OriginPro 2021 9.8.0.200), the DOM content obtained in Table 2 was mapped to obtain Figure 2. The color boxes indicated different pretreatment methods. Red, green and purple boxes represented ultrapure water treatment, ultrapure water treatment followed by sodium pyrophosphate lye treatment, and direct sodium pyrophosphate lye treatment, respectively.

As can be seen from Figure 2A, TOC contents of EPE-MPs, EPS-MPs, FPUF-MPs, RPUF-MPs and LDPE-MPs after extraction with ultrapure water (UPW group) were 1.73, 1.45, 1.40, 1.08 and 1.00 mg/L, respectively. The results of direct extraction with sodium pyrophosphate lye (Pyrophosphate group) were 4.37, 4.09, 3.85, 2.46 and 2.07 mg/L, respectively. TOC content of the Pyrophosphate group was higher than that of the UPW group, indicating that the extraction effect of sodium pyrophosphate lye was better than that of ultrapure water. In addition, the five microplastic fragments treated with ultrapure water were extracted with 20 mL sodium pyrophosphate lye, and the TOC contents in the extracted solution were 2.85, 2.74, 2.52, 1.84 and 1.04 mg/L, respectively. The TOC content of this group was also higher than that of the UPW group, demonstrating that the TOC in microplastics could not be completely extracted with ultrapure water as the extraction agent. In the control group, the TOC contents of water samples in the microplastics sampling sites were 2.75, 2.39, 2.73, 3.07 and 2.42 mg/L, respectively, demonstrating that the difference in water quality of the microplastics sampling sites was negligible. The TOC content adsorbed on RPUF-MPs and LDPE-MPs was lower than that in the control group, making clear that EPE-MPs, EPS-MPs and FPUF-MPs would enrich TOC to a certain extent. The above results showed that the morphology of microplastics affected the adsorption of TOC, and the more abundant the pores, the easier the adsorption of TOC.

Figure 2 is a visual display of material-dependent variability in DOM release. As presented in Figure 2, EPE-MPs and EPS-MPs are high-risk polymers. EPE-MPs consistently released the highest TOC under all treatments (e.g., 4.37 mg/L with pyrophosphate), likely due to their porous, expanded structures providing large surface areas for organic matter adsorption. EPS-MPs followed closely (e.g., 4.09 mg/L TOC), indicating similar vulnerability to chemical treatments. LDPE-MPs showed the lowest TOC release across treatments (e.g., 1.00–2.07 mg/L), attributed to their hydrophobic, non-porous surfaces limiting interactions with water or chelating agents. It is notable that the TOC content adsorbed on RPUF-MPs and LDPE-MPs was lower than that in the control group, suggesting the morphology of MPs would affect the adsorption of TOC, and the more abundant the pores, the easier the adsorption of TOC.

As can be seen from Table 2, the content of IC in the control group was much higher than that of TOC, reaching 16.32, 17.45, 13.97, 14.14 and 14.54 mg/L, respectively, which was 4–6 times that of TOC. As can be seen from Figure 2B, the IC measured by EPE-MPs, EPS-MPs, FPUF-MPs, RPUF-MPs and LDPE-MPs in the UPW group was 12.36, 12.73, 10.26, 8.79 and 5.33 mg/L, respectively. In the Pyrophosphate group, the results were 23.03, 20.32, 18.77, 17.89 and 7.43 mg/L, respectively, making known that the extraction effect of sodium pyrophosphate lye was better than that of ultrapure water. In addition, the five microplastic fragments treated with ultrapure water were extracted with 20 mL sodium pyrophosphate lye, and the measured IC contents were 12.73, 10.37, 9.77, 10.08 and 2.32 mg/L, respectively, indicating that the IC in microplastics could not be completely extracted by using ultrapure water as the extraction agent. It also further showed that the extraction effect of sodium pyrophosphate lye was better than that of the ultrapure water group, and its rule was consistent with TOC.

Based on the summary of Table 2 and Figure 2, the content of DOM (including TOC and IC) adsorbed on microplastics was influenced by the form and material of microplastics, among which the form was the main influencing factor. Microplastics with more pores are more likely to adsorb DOM. There are a large number of pores on the surface of EPE-MPs pearl cotton, and the TOC and IC adsorbed on it were the highest, which was more than 1.5 times that of the surrounding water. The LDPE-MPs film with smooth surface and minimal porosity has the least enrichment effect on DOM, and the TOC content is basically the same as that of the surrounding water, while the IC content is only half of it, expressing that TOC is more easily adsorbed on the LDPE-MPs film than IC. With sodium pyrophosphate lye solution and ultrapure water as the extractor of DOM, the extraction and separation effect of sodium pyrophosphate lye solution was much better than that of ultrapure water, and DOM could be effectively extracted from microplastics in the water environment.

#### 3.1.2. Extraction Methods of DOM from Soil and Beach Sediments

Five soil and five beach sediment samples were conducted in parallel three times according to the above method, and the average value was taken to obtain the TOC and IC contents of the soil and beach sediment extraction solution; meanwhile, seawater around the beach sediment samples was directly taken for testing. The results are shown in Table 3. The optimal extraction method was compared and determined.

**Figure 3:**
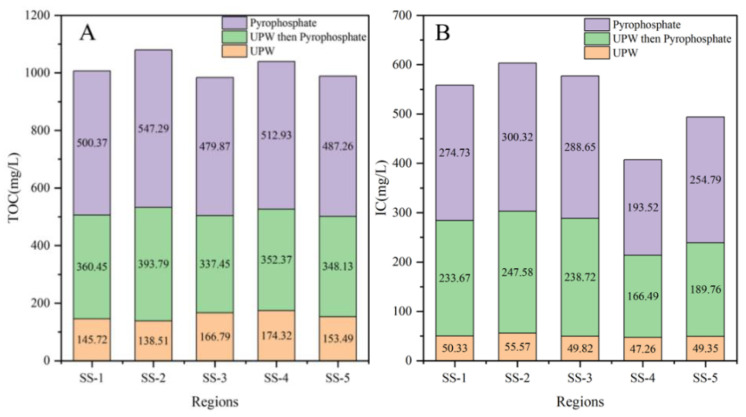

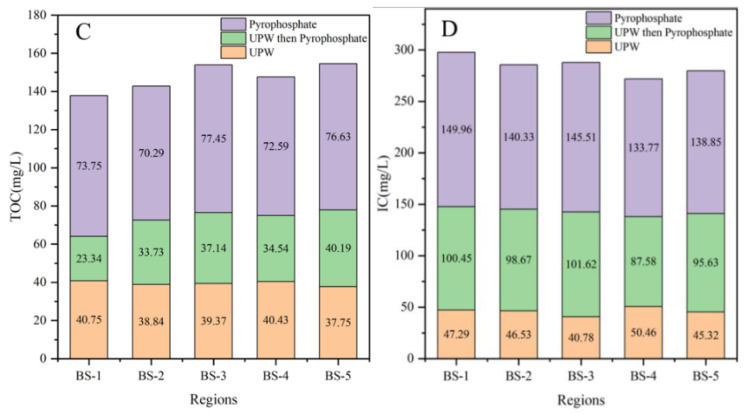
Results of DOM in soil and beach sediments under different pretreatment, where (**A**) and (**C**) represent the intrinsic total organic carbon (TOC) content of soil and beach sand, respectively, whereas (**B**) and (**D**) correspond to their inherent inorganic carbon (IC) content.

The TOC (A and C) and IC (B and D) concentrations contained in the soil and beach sediments obtained in Table 3 were plotted, as shown in Figure 3, where the red, green and purple boxes represented UPW group, UPW then Pyrophosphate group and Pyrophosphate group, respectively.

As can be seen from Figure 3A, soil sediments were directly extracted with sodium pyrophosphate lye, TOC of which were 500.37, 547.29, 479.87, 512.93 and 487.26 mg/L, respectively. It was much higher than that of 145.72, 138.51, 166.79, 174.32 and 153.49 mg/L extracted directly by ultrapure water. However, TOC of soil samples extracted from ultrapure water could still be extracted to 360.45, 393.79, 337.45, 352.37 and 348.13 mg/L, respectively, by using sodium pyrophosphate lye. Combined with Figure 3A,B, it could be seen that TOC content in soil sediments was about 2–3 times higher than IC, and the extraction efficiency of IC by extractants was similar to TOC, indicating that sodium pyrophosphate lye could effectively extract TOC and IC from soil.

Figure 3C showed that beach sediments were directly extracted from sodium pyrophosphate lye with TOC concentrations of 73.75, 70.29, 77.45, 72.59 and 76.63 mg/L, respectively. It was higher than that of 40.75, 38.84, 39.37, 40.43 and 37.75 mg/L for the UPW group. However, TOC of beach sediments treated with ultrapure water and then with sodium pyrophosphate lye were 23.34, 33.73, 37.14, 34.54 and 40.19 mg/L, respectively. TOC concentration of the corresponding seawater was 1/15–1/10 of the above three groups, which were 7.09, 8.32, 7.13, 5.76 and 7.14 mg/L, respectively. Combined with Figure 3C,D, it could be seen that TOC content in beach sediments was small, about half of IC content. The extraction efficiency of ultrapure water for TOC was higher than that for IC, but it was 2–4 times lower than that of the UPW then Pyrophosphate group.

As can be seen from Figure 3, the extraction rate of DOM from beach sediments by ultrapure water was higher than that of soil, but lower than that of sodium pyrophosphate lye solution. Sodium pyrophosphate lye solution can effectively extract TOC and IC from soil and beach sediments.

#### 3.1.3. Extraction Methods of DOM from Microplastics in Soil and Beach Sediments

Five types of microplastics, EPE-MPs, EPS-MPs, FPUF-MPs, RPUF-MPs and LDPE-MPs, collected from the soil and beach sediment (SS-1 to SS-5 and BS-1 to BS-5 in Table 1), were, respectively, used as extractants with ultrapure water and sodium phosphate lye. The results are shown in Table 4.

DOM concentration obtained in Table 4 was plotted, as shown in Figure 3, where the red, green and purple boxes represented UPW group, UPW then Pyrophosphate group and Pyrophosphate group, respectively.

**Figure 4:**
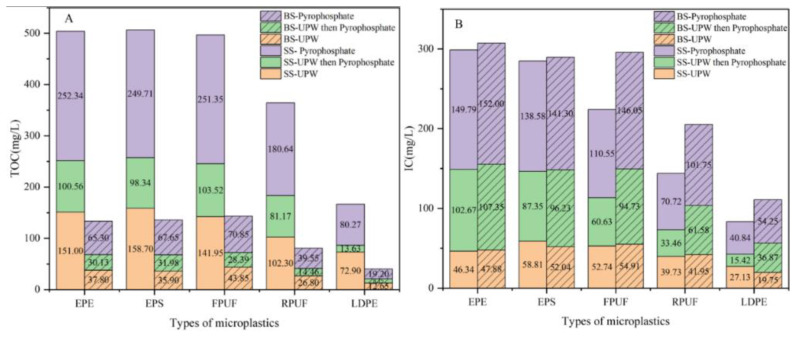
DOM concentrations in soil and beach sediments, where (**A**) and (**B**) are TOC and IC contents, respectively.

From the TOC content in Figure 4A, it could be seen that the extraction efficiency of sodium pyrophosphate lye for DOM was 1.1–1.8 times that of ultrapure water, while for IC, it was 1.5–3.5 times, indicating that IC was more likely to adsorb on microplastics with more voids than TOC. As could be seen from Figure 3A,C and Figure 4A, TOC content in soil itself was about 480–550 mg/L, while TOC content in soil microplastics was up to 252.34 mg/L, accounting for only about half of the TOC of soil itself, demonstrating that TOC cannot be completely adsorbed on microplastics in soil medium. However, for the beach sediment, TOC contained in the beach sand itself was 70–78 mg/L, and the TOC in the beach microplastics was up to 70.85 mg/L, which was similar. For TOC content of microplastics in soil and beach sediments, in addition to the microplastic morphology affecting the adsorption effect, environmental media also influenced the adsorption of TOC, meaning that TOC was more likely to be adsorbed on microplastics in water or flowing media. The microplastics in beach sediments were immersed in seawater for a long time, which made TOC in seawater more easily adsorbed on porous microplastics such as EPE-MPs, EPS-MPs and FPUF-MPs.

It could be seen from Figure 3B,D and Figure 4B that the IC content in soil itself was about 200–300 mg/L, while that in soil microplastics was up to 149.79 mg/L, accounting for about 0.5–0.75 times of the IC concentration in soil itself, indicating that IC could not be completely adsorbed on microplastics in soil. But the adsorption effect was stronger than TOC. IC content of beach sediments itself was 130–150 mg/L, while the IC of beach microplastics reached 54.25–152.00 mg/L. Almost all the IC in beach sand was adsorbed to EPE-MPs, EPS-MPs and FPUF-MPs. However, RPUF-MPs and LDPE-MPs only adsorb part of IC. IC in soil and beach sediments was more easily adsorbed to porous microplastics than TOC.

In summary, for the microplastics in soil and beach sediments, not only the morphology of microplastics would affect the adsorption effect, but also the environmental media would affect the adsorption of DOM.

### 3.2. DOM Content in Microplastics with Different Environmental Media

The microplastics collected from different environmental media in Table 1 were extracted with sodium pyrophosphate lye as the extraction agent. The extraction methods were carried out according to the methods listed in Section 2.3, and TOC and IC contents were determined to obtain Table 5.

**Figure 5:**
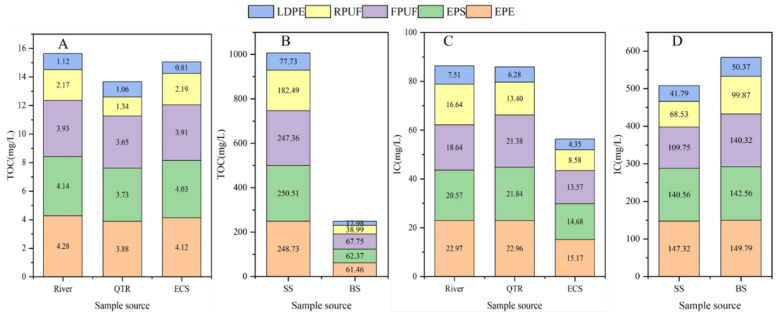
DOM concentrations of MPs in different environmental media, where (**A**,**B**) and (**C**,**D**) were TOC and IC concentrations, respectively.

DOM concentrations of microplastics in different environmental media obtained in Table 5 were analyzed, and Figure 5 was obtained, where (A–B) and (C–D) were TOC and IC concentrations, respectively.

It could be seen from Figure 5A,B that TOC content adsorbed on microplastics of river, QTR and ECS was between 0.81–4.28 mg/L, and TOC adsorbed on EPE-MPs, EPS-MPs and FPUF-MPs was 1.67–5.28 times that of RPUF-MPs and LDPE-MPs, indicating that EPE-MPs, EPS-MPs and FPUF-MPs would further enrich TOC. However, TOC content in soil microplastics varied greatly among different types of microplastics, reaching 247.36–250.51 mg/L on EPE-MPs, EPS-MPs and FPUF-MPs, 182.49 mg/L on RPUF-MPs, but only 77.73 mg/L on LDPE-MPs. Although the TOC concentration in soil microplastics was much higher than that in water environment microplastics, it was still less than 1/2 of the TOC contained in the soil itself. The DOM content of beach microplastics was similar to that of soil microplastics, and EPE-MPs, EPS-MPs and FPUF-MPs would further enrich TOC, which was 8.78–9.68 times that of the corresponding seawater sample (about 7.0 mg/L), with obvious enrichment function. The enrichment capacity of LDPE-MPs was the worst, even reaching 2.54 times, which further indicated that the moist and humid environmental medium was more conducive to the adsorption of TOC in microplastics. The variation of IC in microplastics of water environment, soil and beach sediments showed the same pattern as that of TOC.

### 3.3. Morphology Characterization of Microplastics Before and After Extraction

The adsorption and desorption behavior of microplastics on dissolved organic matter (DOM) is affected by a variety of environmental factors, including pH, salinity and temperature. The value of pH influences the surface charge of MPs and DOM, changes the molecular structure and functional group ionization state of DOM, and thus impresses the interaction between MPs and DOM [49]. At low pH, the surface of MPs is positively charged, and easy to adsorb the negatively charged DOM. At high pH, the surface of microplastics has negative charge, which decreases the adsorption capacity and results in the easy resolution of DOM [50]. High salinity amplifies the ionic strength of the solution, compresses the double electric layer, weakens the electrostatic repulsion between MPs and DOM, and elevates adsorption [51,52]. However, low salinity lowers the adsorption of MPs to DOM and intensifies desorption. High temperature expands the molecular motion, which may enhance the adsorption rate, but may also decline the adsorption bond strength and strengthen desorption. High temperatures generally elevate DOM solubility and reduce its adsorption on MPs surfaces [53]. Low temperature generally diminishes the adsorption of MPs to DOM [54].

**Figure 6:**
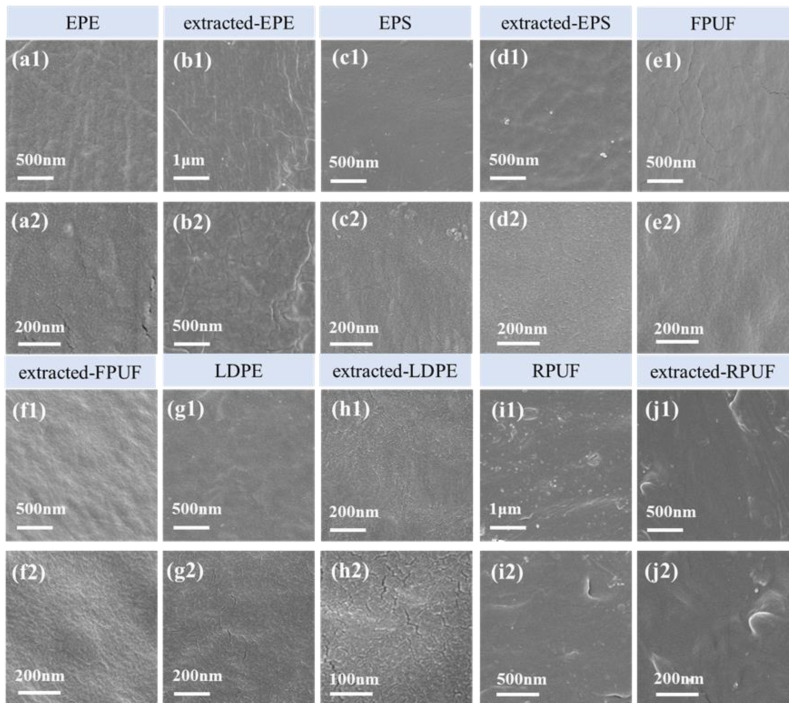
SEM morphology characterization of 5 different types of microplastics before and after extraction. Panels (**a1**) and (**a2**) display SEM of EPE microplastics at distinct magnifications, while (**b1**) and (**b2**) show sodium pyrophosphate-extracted EPE microplastics under corresponding scales. Similarly, (**c1**,**c2**) and (**d1**,**d2**) present SEM of EPS-MPs and their sodium pyrophosphate-extracted counterparts, respectively. FPUF and LDPE microplastics are depicted in (**e1**,**e2**) and (**g1**,**g2**), with their post-extraction morphologies illustrated in (**f1**,**f2**) and (**h1**,**h2**), respectively. Lastly, RPUF microplastics and their sodium pyrophosphate-treated variants are visualized in (**i1**,**i2**) and (**j1**,**j2**) at varying magnifications.

The scanning electron microscopy (SEM) images (Figure 6) of five kinds of microplastics before and after extraction with sodium pyrophosphate-NaOH were introduced.

SEM showed that the appearance of the five microplastics did not change significantly before and after the extraction of sodium pyrophosphate-NaOH. Before extraction, the surface of the five kinds of microplastics was smooth, uniform and dense, and there were no obvious cracks, pores and pits. After extraction, the surface properties and structures of the five kinds of microplastics remained stable, and there were no obvious changes in morphology or chemical properties such as cracks and pores, indicating that the surface chemical properties and physical forms of the five kinds of microplastics showed high stability in the low temperature and short-term strong alkali solution.

### 3.4. Molecular Weight Distribution of DOM in Microplastics with Different Environmental Media

DOM with molecular weight between 400–1000 Da is mainly composed of small organic compounds such as aromatic, fatty, and amino acids, monosaccharides and oligosaccharides. DOM in the molecular weight range of 1000–2000 Da is mainly made up of protein, polysaccharide degradation products, aromatic compounds and microbial metabolites. DOM with molecular weights ranging from 2000 to 3000 Da mainly consists of long chain unsaturated fatty acids, amino acids, plant extracellular polysaccharides and other small organic molecules. DOM with a molecular weight of 3000–5000 Da mainly contains complex substances, such as polysaccharides, protein fragments, fulvic acid and humic acid, which usually have a high degree of humification and may be derived from terrigenous or microbial activities. DOM with a molecular weight range of 5000–7000 Da is mainly constituted by complex organic substances such as fulvic acid and humic acid.

The molecular weight of DOM as shown in Table 5 was determined by GPC analyzer. In order to further study the differences of DOM adsorption by different kinds of microplastics, DOM can be divided into high, middle and low molecular regions according to weight-average molecular weight (*M_w_*). In the GPC spectrum, DOM can be divided into high, medium and low molecular regions according to the *M_w_*, in which small molecular region with *M_w_* less than 1000 Da, middle molecular region with *M_w_* 1000–10,000 Da and the polymer region with *M_w_* greater than 10,000 Da.

Calibration with the standard glucan series with molecular weights of 180, 342, 505, 1300, 2800, 5000, 12,000, 25,000, 49,600 and 62,900 Da, the standard equation was Log Mol W*_t_* = 2.67 × 10^1^ – 7.72 × 10^0^ T1 + 8.77 × 10^−1^ T2 – 3.38 × 10^−2^ T3, R^2^ = 0.999114, indicating better fitting of standard curve. It was suitable and feasible to use dextran with different molecular weight as correction curve to predict DOM molecular weight, and the method has good repeatability.

**Figure 7:**
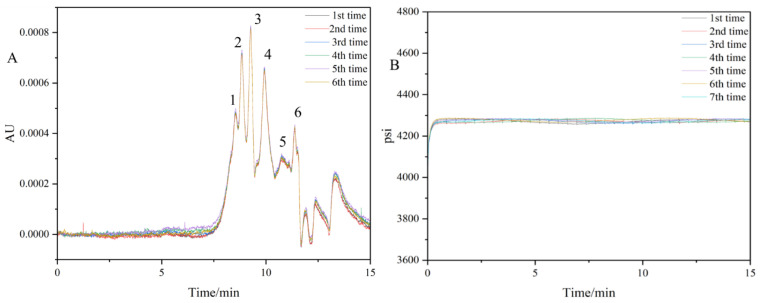
Repeated exclusion chromatogram (**A**) and sample pressure repeatability diagram (**B**) of River-1^a^ sample.

Although the high molecular weight protein in DOM was less, it was also easy to adsorb in the pipeline and column of GPC chromatography, resulting in poor repeatability of peak area. The DOM obtained from River-1^a^ microplastics was injected for six consecutive times to obtain the six-times repeatable GPC chromatography shown in Figure 7A. DOM was a complex mixture that was difficult to separate chromatographically. The repetitive chromatogram showed that although the baseline was not completely separated, it could still be quantitatively and qualitatively detected. It had six peaks, which were called peak 1 to peak 6 in descending order of molecular weight. Molecular weight can be divided into *M_w_*, number average molecular weight (*M_n_*) and Z average molecular weight (*M_z_*). The relative standard deviation (RSD) of six peaks in these three molecular weights were all lower than 2%, meaning that the data of each group were close to the mean value and the method had good reproducibility. The molecular weight distribution of DOM in River-1^a^ microplastics showed a multi-peak pattern, and the *M_w_* corresponding to the 6 peaks were 5372, 4541, 3955, 2771, 1169 and 421 Da, respectively. The peaks of 6 samples have good overlap and good repeatability can be obtained. The 6-times repeatable chromatogram showed good overlap of peaks, demonstrating good repeatability. In order to investigate the stability of the GPC analyzer, blank ultrapure water was used for 7 consecutive times to test the pressure repeatability, and the sample pressure reproducibility diagram in Figure 7B was obtained. It could be seen from the pressure reproducibility diagram that the analyzer had good stability and repeatability, and the scheme of measuring DOM molecular weight distribution by GPC analyzer was feasible.

**Figure 8:**
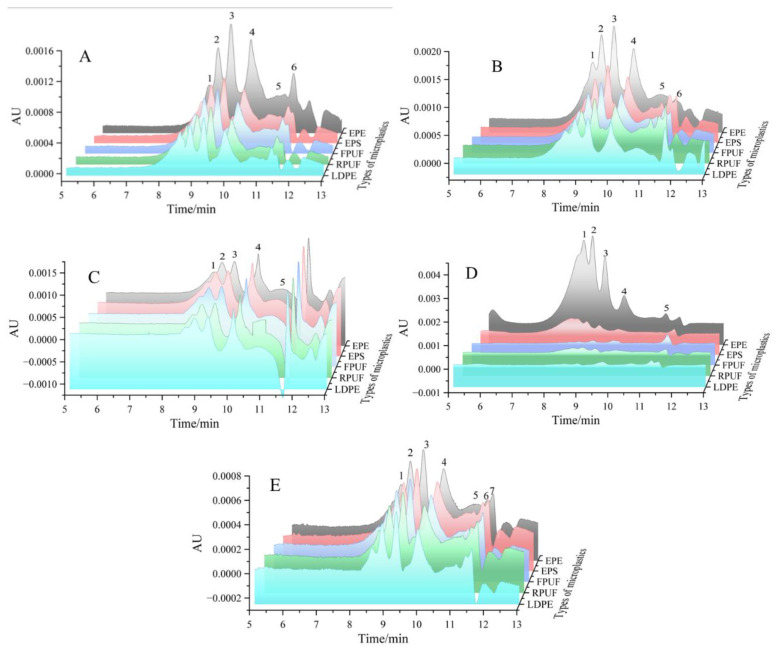
3D map of DOM molecular weight distribution adsorbed on five kinds of microplastics in different environmental systems, where (**A**–**E**) represented river, Qiantang River, East China Sea, soil and beach sediments, respectively.

The molecular weight of DOM adsorbed on five kinds of microplastics collected from River, QTR, ECS, SS and BS was detected. The peak and peak value of the outflow were shown in Table 6, and the three-dimensional curve was shown in Figure 8. In Figure 8, the horizontal coordinate represented the retention time, and the vertical coordinate depicted the peak signal intensity. According to the order of molecular weight from large to small, the molecules were arranged and separated from the GPC column successively with high molecular weight peaks first, followed by low molecular weight peaks. The signal symbolized the target, and in general, several peaks indicated molecules containing several molecular weight distributions. The signal strength represented the molecular content, and the greater the intensity, the higher the molecular content, and vice versa. The molecular weight corresponding to the peak could be obtained by the time of the outflow curve combined with the conversion of the correction curve. As shown in Figure 7, the molecular weight distribution of DOM adsorbed by microplastics in different environmental systems expressed a multi-modal distribution pattern.

DOM in water environment microplastics was different from that in soil and beach sediment microplastics. From the molecular weight distribution of DOM obtained by River^a^ microplastics in Figure 8A, it could be seen that different microplastics had different adsorption properties for DOM, in which EPE-MPs > EPS-MPs > FPUF-MPs > RPUF-MPs > LDPE-MPs, and the peak emergence of DOM was slightly different, but not significant. The molecular weight distribution was multi-peak, each DOM contained six peaks, numbered from left to right, respectively, peak 1 to peak 6, and the corresponding *Mw* was 5288–5489, 4522–4552, 3910–3986, 2750–2788, 1171–1267 and 401–412 Da. The molecular weight was mainly distributed in peak 2 to peak 4, that is, *M_w_* of the DOM above was between 2750–4552 Da, mainly fulvic acid and humic acid, with a small part of unsaturated long-chain fatty acids, amino acids and plant extracellular polysaccharides.

As shown in Figure 8B, the DOM adsorption capacity of microplastics in Qiantang River was roughly the same as that in river water, and the adsorption capacity was EPE-MPs > EPS-MPs > FPUF-MPs > RPUF-MPs > LDPE-MPs. The DOM, peak 3 of EPE-MPs in Qiantang River was the highest, reaching 0.002 AU, which was slightly higher than the 0.0016 AU of EPE-MPs in river water. The molecular weight of DOM in the five kinds of microplastics from Qiantang River showed multi-peak distribution, and the retention time and molecular weight distribution of the first four peaks had little difference, and the corresponding *Mw* were 5327–5402, 4518–4557, 3867–3972 and 2628–2760 Da, respectively. It was mainly composed of amino acids, fulvic acids and humic acids.

It could be seen from Figure 8C that the DOM adsorption performance of five different kinds of microplastics in the East China Sea was EPE-MPs > EPS-MPs > FPUF-MPs > RPUF-MPs > LDPE-MPs, and the molecular weight of DOM was distributed in a multi-peak manner. In addition to the differences in the content of DOM, the retention time and molecular weight of the first five peaks were little different. The corresponding *Mw* was 5203–5228, 4465–4540, 3888–3914, 2498–2530 and 1123–1165 Da, respectively. The molecular weight of the five DOM species in ECS microplastics was mainly distributed between peak 1 and peak 5 (1123–5228 Da). Except for amino acids, fulvic acid and humic acid, DOM also contained a certain amount of protein and polysaccharide degradation products, aromatic compounds and other moderate molecular weight compounds.

As shown in Figure 8D, the DOM absorption on five kinds of microplastics in the soil sediments was EPE-MPs >> EPS-MPs > FPUF-MPs ≈ RPUF-MPs > LDPE-MPs, where the DOM content adsorbed on EPE-MPs was about seven times that of EPS-MPs, two times that of FPUF-MPs, and 17.5 times that of LDPE-MPs. The DOM adsorbed by five kinds of microplastics in soil sediments was very different, and the peak retention time and *Mw* were significantly different, and the DOM was obviously distinct from that in river, Qiantang River, East China Sea and other water environments. The *M_w_* of DOM in EPE microplastics was foremost distributed in peak 1 to peak 4, which were 6190, 4780, 4148 and 3051 Da, respectively. The *Mw* of DOM in EPS microplastics was also primarily distributed in peak 1 to peak 4, and its *M_w_* was 7302, 5360, 4933 and 4305 Da, respectively, which was prominently different from that in EPE microplastics. The *Mw* of DOM in FPUF microplastics was dominantly distributed in peak 2 to peak 5, which were 4313, 3299, 1731 and 440 Da, respectively. The average *Mw* represented by each peak differed from that in EPE and EPS microplastics, which was smaller *M_w_*. The *M_w_* of DOM was 5179, 4520, 3940 and 2756 Da for RPUF-MPs, and 4466, 3215, 1701 and 1671 Da for LDPE-MPs, respectively.

According to Figure 8E, the DOM absorption of the five kinds of microplastics in beach sediments could be observed as EPE-MPs > EPS-MPs >> FPUF-MPs >> RPUF-MPs > LDPE-MPs. The content of DOM in EPE-MPs was about 2 times, 10 times, 26 times and 52 times that of EPS-MPs, FPUF-MPs, RPUF-MPs and LDPE-MPs, respectively. The *Mw* of DOM in beach sediment microplastics was primarily distributed in 5234–6178, 4358–4790, 3325–4153, 1697–3047 and 438–1163 Da, which were principally complex compounds such as polysaccharides, protein fragments, folic acid and humic acid derived from terrigenous or microbial activities.

### 3.5. Fluorescence Characteristics of DOM in Microplastics with Different Environmental Media

**Figure 9:**
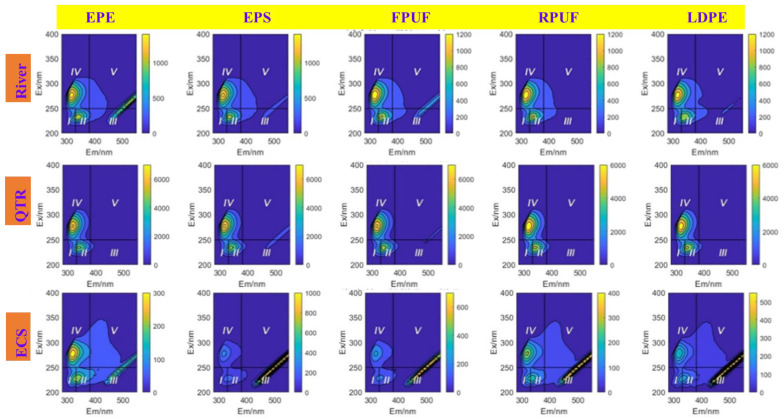

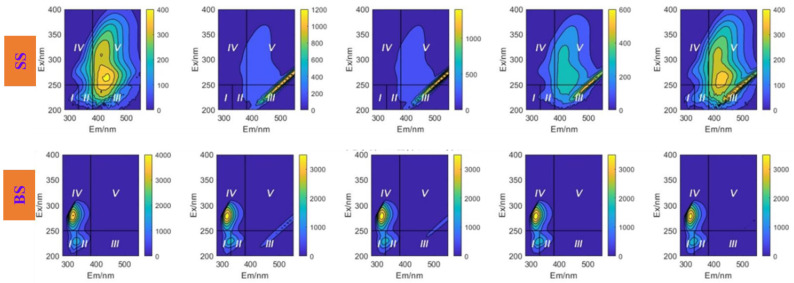
3D fluorescence spectra of 25 DOM.

Three-dimensional fluorescence analysis of DOM obtained from five groups of microplastics with diverse environmental media was performed, and Figure 9 was obtained.

As shown in Figure 9, the three-dimensional fluorescence characteristics of 25 kinds of DOM revealed striking differences, which meant that the fluorescence peak type and fluorescence intensity changed with the variation of environmental media and microplastics. By observing Figure 9, DOM onto microplastics from River, QTR and ECS all contained protein-like and soluble microbial byproducts C1 in zone IV (Ex/Em = 275 nm/315 nm, highly stimulated tryptophan-like fluorescent substance), aromatic protein-like C2 in zone II (Ex/Em = 230–235 nm/335–340 nm), and aromatic protein-like C3 in region I (Ex/Em = 280–300 nm/330–350 nm, low excitation light tyrosine-like fluorescent substance). C1, C2 and C3 showed obvious fluorescence peaks. Obvious fluorescence peaks appeared in C1, C2 and C3, while an almost invisible fluorescence peak illustrated in humic-like C4 in zone V, manifesting that the humic-like content was low in the river, QTR and ECS microplastics, and the soluble microbial byproducts content was the highest, followed by the aromatic protein substances. From the type and intensity of fluorescence peaks, it also made clear that microbial activities were very active in the water environment, and microorganisms produce soluble microbial by-products by decomposing organic matter. Low humic-like content might mean that a large part of the refractory organic matter had been degraded by microorganisms, the degree of degradation of organic matter was higher, and might be affected by specific environmental conditions. The current environment may be more conducive to microbial growth and metabolism than humus accumulation. Interestingly, extremely weak fluorescence peak C4 (zone V, Ex/Em = 325 nm/415 nm) of marine humic acid was also detected in the DOM of five ECS microplastics, which indicates that DOM adsorbed on the ECS microplastics may have a high endogenous level, resulting from endogenous biodegradation, while the fluorescence peak of Marine humic acid was usually related to exogenous terrestrial input. In addition, changes in salinity will also affect the position and intensity of the fluorescence peak of DOM adsorbed on microplastics, especially in low salinity, the fluorescence peak of marine humic acid may weaken or disappear, so that the fluorescence peak is not obvious.

**Figure 10:**
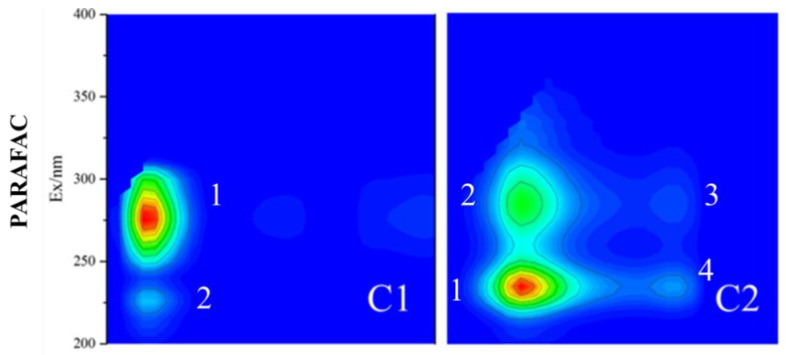
PARAFAC analysis of 25 DOM adsorbed by microplastics.

Parallel factor analysis (PARAFAC) was used to analyze the DOM adsorbed by the above 25 microplastics and resolve the adsorbable fluorescence components on different environmental media and types of microplastics. The PARAFAC results were shown in Figure 10.

Figure 10 could characterize the main organic types of DOM in microplastics with 25 different environmental media. PARAFAC showed that C1 and C2 were two main fluorescent substances, in which C1 and C2 had two and four fluorescence peaks, respectively. C1 had two fluorescence peaks, of which peak 1 belonged to dissolved microbial metabolites in zone IV, mainly tyrosine-like and protein-like organic matter (Ex/Em = 275 nm/315 nm), which was mainly organic matter produced by natural decomposition of microorganisms, algae, aquatic higher plants in water, and pertained to endogenous DOM, contributing the main part. However, exogenous input also contributed to a part. DOM of the terrestrial ecosystem entered the water body through exogenous processes such as rainfall and runoff. Peak 2 of C1 was the tyrosine organic compound in zone I (Ex/Em = 230 nm/315 nm), which was associated with the class of aromatic protein I. The different fluorescence intensity may reflect the characteristics of diverse components or distinct molecular structures in the sample. The fluorescence intensity of peak 1 for C1 was much higher than that of peak 2, which meant that the organic component corresponding to peak 1 had a higher concentration or quantum yield in the sample, or higher excitation efficiency, and the corresponding compound had a smaller molecular weight. The short excitation wavelength of peak 2 demonstrated implicitly that its excitation required higher excitation energy, such as aromatic ketones in the water environment. The compound corresponding to peak 2 had higher bond energy, molecular stability, and larger molecular weight, which could effectively react with the energy transfer of pollutants, thus promoting the photodegradation of pollutants. The excitation wavelength (Ex) of C1 had two different excited states, and these excited states merged into one major emission peak when emitted, which was called “multiple excited states” or “multiple emission peaks” in spectroscopy. The possible reason for this phenomenon was that C1 of DOM adsorbed on microplastics had two excited states, but only showed a major emission peak at the time of emission due to energy transfer or recombination. “Multiple excited states” have important research value in spectroscopy, which can help us better understand the molecular structure and electronic transition mechanism of DOM.

C2 had four fluorescence peaks, of which peak 1 was the II class of aromatic protein composed of aromatic protein substances such as aromatic amino acids (Ex/Em = 235 nm/340 nm), mainly from the metabolites of phytoplankton and microorganisms in the water, belonging to the endogenous DOM. Peak 2 was tryptophan proteins of soluble microbial by-products in zone IV (Ex/Em = 280 nm/340 nm), which might contain tryptophan, tyrosine, phenylalanine and other amino acid residues containing conjugated double bonds. The fluorescence intensity and molecular weight of the compound corresponding to peak 1 were greater than that of peak 2. Peak 3 (Ex/Em = 235 nm/420 nm) and peak 4 (Ex/Em = 285 nm/420 nm) were part of the fulvic-like fluorescent substances located in region III and V, respectively. The fluorescence peaks of peak 3 and peak 4 were faintly visible, indicating that the content of fluorescent substances contained in peak 3 and peak 4 was less or there might be other influencing factors, leading to the overlap or interference of fluorescence peaks.

## 4. Conclusions

Sodium pyrophosphate-NaOH solution, as an extraction agent, can effectively extract DOM adsorbed on microplastics from different environmental media such as river, Qiantang River, seawater, soil and beach sediments. Sodium pyrophosphate is a strong alkaline solution with high solubility and ionic strength. Studies had shown that sodium pyrophosphate can effectively extract nanoparticles from soil without significantly changing the physical and chemical properties of the particles [55]. The adsorption behavior of sodium pyrophosphate on biochar was consistent with the Langmuir isotherm model, indicating that it was mainly adsorbed by physical adsorption [56]. In addition, sodium pyrophosphate may enhance the DOM adsorption capacity by changing the charge distribution on the surface of microplastics or forming complexes [57]. This was because of the non-depolymerizable hydrolysis of sodium pyrophosphate (Na_4_P_2_O_7_) in water. It had four stages of hydrolysis, forming HP_2_O_7_^3−^, H_2_P_2_O_7_^2−^, H_3_P_2_O_7_^−^, and H_4_P_2_O_7_ respectively. In the condition of NaOH solution, the pyrophosphate ion (P_2_O_7_^4−^) readily depolymerized. When pH was 13, the contents of phosphate and hydrogen phosphate were 83% and 17%, respectively [58], which were dominated by phosphate ions. The key role of sodium pyrophosphate-NaOH solution in the extraction of DOM adsorbed by microplastics was phosphoric acid ion after hydrolysis. The extraction mechanism of phosphoric acid ion and DOM was a complex process, involving ion exchange, complexation, adsorption, microbial metabolism and environmental factors.

The form, type and environmental medium of microplastics were the main influencing factors of DOM adsorption on microplastics. For microplastics in water environment, the morphology was the principal factor affecting DOM adsorption. The more abundant microplastics pores were, the easier DOM adsorption was, and the adsorption capacity of TOC and IC on them was roughly the same. The DOM content adsorbed on EPE-MPs, EPS-MPs and FPUF-MPs was 2–3 times that of DOM in water environment, among which EPE-MPs pearl cotton with the most porosity had the highest DOM content adsorbed on it, while LDPE film with smooth surface and the least pores had the least DOM enrichment effect, about 0.5–1 times. For microplastics in soil and beach sediments, microplastics morphology and environmental media were the two leading factors affecting DOM adsorption. DOM was more likely to be adsorbed on microplastics in flowing media, meanwhile the microplastics in beach sediment were immersed in seawater for a long time, resulting in DOM in seawater being more likely to be adsorbed on porous microplastics such as EPE-MPs, EPS-MPs and FPUF-MPs. In non-flowing soil media, the DOM content adsorbed on microplastics was less than 50% of the DOM contained in soil, which further indicates that DOM in soil could not be completely adsorbed on microplastics in non-flowing media.

The molecular weight distribution of DOM adsorbed by microplastics in different environmental systems showed a wide molecular weight distribution and multi-modal distribution pattern, containing both low molecular weight polymers and high molecular weight polymers, indicating that the DOM source composition was complex. In the water environment, the DOM adsorption capacity of five different types of microplastics was EPE-MPs >EPS-MPs >FPUF-MPs >RPUF-MPs >LDPE-MPs from large to small. The enrichment ratio of EPE-MPs, EPS-MPs and FPUF-MPs for DOM in water environment was about 1.5–3 times, while that of RPUF-MPs and LDPE-MPs was only 0.5–1 times. The adsorption capacity of microplastics to DOM in soil and beach sediments differed greatly from that in water environment. The absorption of soil sediment microplastics to DOM was EPE-MPs >> EPS-MPs > FPUF-MPs ≈ RPUF-MPs > LDPE-MPs, which was EPE-MPs > EPS-MPs >> FPUF-MPs ≫ RPUF-MPs > LDPE-MPs in beach sediment microplastics. The molecular weight distribution of DOM adsorbed by five kinds of microplastics in soil and beach sediments was significantly different, and the DOM adsorbed by EPE-MPs, EPS-MPs and RPUF-MPs was more distributed in the high molecular weight (>2500 Da), while FPUF-MPs and LDPE-MPs were more distributed in the low molecular weight (<2500 Da).

By PARAFAC, the DOM adsorbing on 25 microplastics contained two principal components, accounting for 37.4% of C1 and 62.6% of C2, respectively. C1 contained highly excited tyrosine-like fluorescent substances in zone IV (Ex/Em = 275 nm/315 nm) and low excited tyrosine-like fluorescent substances in zone I (Ex/Em = 230 nm/315 nm), which had the strongest adsorption capacity on microplastics. C2 involved low excitation tryptophan-like fluorescent substances in zone II (Ex/Em = 235 nm/340 nm) and protein-like tryptophan (Ex/Em = 280 nm/340 nm) fluorescent substances in zone IV, which had the second adsorption capacity in microplastics and came from the degradation metabolites of phytoplankton and microorganisms. C2 also embraced visible humic acid fluorescent substances in zone V (Ex/Em = 280 nm/420 nm) and ferric acid fluorescent substances in zone III (Ex/Em = 235 nm/420 nm), which had the weakest adsorption capacity in microplastics.

The highly excited tyrosine-like fluorescent substances in the IV region and the low excited tyrosine-like fluorescent substances in the I region had the strongest adsorption capacity on microplastics, which might be caused by the comprehensive effects of hydrophobic, π-π conjugation, hydrogen bond and static electricity. (1) The above two types of fluorescent substances had certain hydrophobicity, and there were usually hydrophobic regions on the surface of microplastics. The more hydrophobic substances were, the more likely they were to accumulate in the hydrophobic region of microplastics through hydrophobic action [59]. (2) The tyrosine-like fluorescent substances in regions IV and I might contain aromatic ring structures, which could further enhance adsorption by interacting with the aromatic ring structures on the surface of microplastics through π-π conjugation [60]. (3) During the aging process, the surface of microplastics would produce more hydroxyl, carboxyl and other oxygen-containing functional groups, which could form hydrogen bonds with polar groups in tyrosine-like fluorescent substances and enhance adsorption capacity [61]. (4) The surface charge of microplastics and fluorescent substances could generate electrostatic interaction, which affected the adsorption efficiency and further improved the adsorption [62]. (5) Distinct morphologies of microplastics resulted in diverse sizes and specific surface areas. Smaller fluorescent substance molecules were more likely to enter the micropores and surface cracks of microplastics, increasing the contact area and thus enhancing the adsorption capacity. In summary, EPE-MPs, EPS-MPs, FPUF-MPs and RPUF-MPs had rich pore structure, and were more likely to adsorb small molecular weight molecules in the highly excited tyrosine-like in the region IV and the low excited tyrosine-like in the region I.

The adsorption of dissolved organic matter (DOM) on different kinds of microplastics provided significant implications for pollution control. As the carrier of DOM in water, microplastics could influence the migration, transformation and environmental fate of DOM, which not only changed the distribution of DOM, but also might improve its bioavailability and toxicity. Therefore, in pollution control, it was extremely necessary to consider the impact of microplastics on DOM behavior in order to more effectively control and treat organic pollutants in water bodies. At the same time, different kinds of microplastics could significantly change the adsorption capacity of DOM, which had a much greater impact on the removal efficiency of pollutants to a certain extent. In addition, the interaction between microplastics and DOM might also have an effect on the health of aquatic ecosystems, and comprehensive measures should be taken to reduce the emission of microplastics in the treatment of environmental pollution, and the removal of microplastics and DOM in water through physical, chemical and biological methods.

The phenomenon of DOM adsorption on microplastics indicates that the interaction between microplastics and DOM and its environmental effects should be comprehensively considered in pollution control, in order to formulate more effective treatment strategies to protect the environment and ecosystem health.

The future research direction can be roughly divided into three directions. The first is to discuss the long-term stability of DOM on microplastics, and study the long-term changes and environmental influencing factors after the combination of DOM and microplastics. Second, it is necessary to explore the physical and chemical effects of DOM-bound microplastics in the food chain, as well as the transmission and cumulative effects of toxicity, bioavailability, and ecosystem function interference. Finally, the regulatory mechanisms of environmental factors (such as ultraviolet radiation and microbial activity) on microplastics-DOM interaction were analyzed. These research directions can not only deepen our understanding of the environmental behavior of microplastics, but also provide a more comprehensive scientific basis for assessing their ecological risks.

## Figures and Tables

**Table 1:** Information of Microplastic Sampling from Different Environmental Media.

Medium	GPS Positioning (Latitude, Longitude)	Types of MPs	Medium	GPS Positioning (Latitude, Longitude)	Types of MPs
River-1 ^a^	30.33040°, 120.39633°	EPE	ECS-4 ^c^	30.33818°, 120.80121°	RPUF
River-2 ^a^	30.33037°, 120.39063°	EPS	ECS-5 ^c^	30.35401°, 120.77329°	LDPE
River-3 ^a^	30.32942°, 120.40831°	FPUF	SS-1 ^d^	30.32634°, 120.46731°	EPE
River-4 ^a^	30.32659°, 120.40932°	RPUF	SS-2 ^d^	30.32999°, 120.46791°	EPS
River-5 ^a^	30.32161°, 120.40679°	LDPE	SS-3 ^d^	30.25458°, 120.29443°	FPUF
QTR-1 ^b^	30.39931°, 120.43359°	EPE	SS-4 ^d^	30.32635°, 120.47169°	RPUF
QTR-2 ^b^	30.38755°, 120.43431°	EPS	SS-5 ^d^	30.33388°, 120.47373°	LDPE
QTR-3 ^b^	30.39329°, 120.43452°	FPUF	BS-1 ^e^	30.71412°, 121.35589°	EPE
QTR-4 ^b^	30.39502°, 120.43779°	RPUF	BS-2 ^e^	30.71301°, 121.35204°	EPS
QTR-5 ^b^	30.38496°, 120.43001°	LDPE	BS-3 ^e^	30.71267°, 121.34392°	FPUF
ECS-1 ^c^	30.38878°, 120.86526°	EPE	BS-4 ^e^	30.73846°, 121.38266°	RPUF
ECS-2 ^c^	30.32326°, 120.84919°	EPS	BS-5 ^e^	30.72296°, 121.36535°	LDPE
ECS-3 ^c^	30.34964°, 120.81228°	FPUF	/	/	/

Note: The symbols ^a^, ^b^, ^c^, ^d^, and ^e^ denote specific sampling locations as follows: (a) corresponds to the inland rivers within Hangzhou Xiasha higher education park, (b) to the Qiantang River section in Haining district, Jiaxing city, (c) to the East China Sea area in Jinshan district, Shanghai, (d) to the soil in Hangzhou Dajiangdong farm, and (e) to the beach area in Jinshan district, Shanghai, respectively.

**Table 2:** The concentration of DOM contained in environmental media (River-1^a^) and microplastics.

Group	Mean of TOC (mg/L)	TC (mg/L)	Mean of IC (mg/L)	Source
Control	2.75 ± 0.10 B	19.07	16.32 ± 0.10 B	River-1 water
	2.39 ± 0.10 C	19.84	17.45 ± 0.16 A	River-1 water
	2.73 ± 0.14 B	16.70	13.97 ± 0.11 C	River-1 water
	3.07 ± 0.11 A	17.21	14.14 ± 0.85 C	River-1 water
	2.42 ± 0.09 C	16.96	14.54 ± 0.12 C	River-1 water
Pyrophosphate	4.37 ± 0.10 A,a	27.40	23.03 ± 0.1 A,a	EPE-MPs
	4.09 ± 0.16 AB,a	24.41	20.32 ± 0.13 B,a	EPS-MPs
	3.85 ± 0.10 B,a	22.62	18.77 ± 0.12 C,a	FPUF-MPs
	2.46 ± 0.17 C,a	20.35	17.89 ± 0.13 D,a	RPUF-MPs
	2.07 ± 0.07 D,a	8.50	7.43 ± 0.09 E,a	LDPE-MPs
UPW	1.73 ± 0.06 A,c	14.09	12.36 ± 0.09 B,b	EPE-MPs
	1.45 ± 0.10 B,c	14.18	12.73 ± 0.17 A,b	EPS-MPs
	1.40 ± 0.06 B,c	11.66	10.26 ± 0.11 C,b	FPUF-MPs
	1.08 ± 0.12 C,c	9.87	8.79 ± 0.12 D,b	RPUF-MPs
	1.00 ± 0.07 C,b	6.33	5.33 ± 0.13 E,b	LDPE-MPs
UPW then Pyrophosphate	2.85 ± 0.11 A,b	15.58	12.73 ± 0.15 A,c	EPE-MPs
	2.74 ± 0.05 A,b	13.11	10.37 ± 0.12 B,c	EPS-MPs
	2.52 ± 0.19 A,b	12.29	9.77 ± 0.12 B,c	FPUF-MPs
	1.84 ± 0.14 B,b	11.92	10.08 ± 0.12 B,c	RPUF-MPs
	1.04 ± 0.11 C,b	3.36	2.32 ± 0.10 C,c	LDPE-MPs

Values expressed as mean ± standard deviation obtained from three measurements. Capital letters indicate significant differences related to different sources in a certain group, while lowercase letters indicate significant differences related to different groups for a certain kind of MPs. For each type of MPs, there were significant differences among the three different treatments, among which pyrophosphate treatment had the highest data, followed by UPW then pyrophosphate, and UPW had the lowest. Notably, for LDPE-MPs, there was no statistically significant difference between UPW then Pyrophosphate and UPW.

**Table 3:** Results of DOM in soil and beach sediments under different pretreatment.

Serial	Mean of TOC (mg/L)	TC (mg/L)	Mean of IC (mg/L)	Sample Type	Source
Pyrophosphate Group	500.37 ± 1.27 C,a	775.1	274.73 ± 1.14 C,a	Soil sediments	SS-1
	547.29 ± 1.10 A,a	847.61	300.32 ± 0.96 A,a	Soil sediments	SS-2
	479.87 ± 1.37 E,a	768.52	288.65 ± 1.09 B,a	Soil sediments	SS-3
	512.93 ± 1.27 B,a	706.45	193.52 ± 0.86 E,a	Soil sediments	SS-4
	487.26 ± 1.58 D,a	742.05	254.79 ± 0.95 D,a	Soil sediments	SS-5
UPW Group	145.72 ± 1.26 D,c	196.05	50.33 ± 1.04 B,c	Soil sediments	SS-1
	138.51 ± 1.19 E,c	194.08	55.57 ± 1.07 A,c	Soil sediments	SS-2
	166.79 ± 1.20 B,c	216.61	49.82 ± 1.05 B,c	Soil sediments	SS-3
	174.32 ± 1.21 A,c	221.58	47.26 ± 1.15 B,c	Soil sediments	SS-4
	153.49 ± 1.30 C,c	202.84	49.35 ± 1.16 B,c	Soil sediments	SS-5
UPW then Pyrophosphate Group	360.45 ± 1.34 B,b	594.12	233.67 ± 1.09 C,b	Soil sediments	SS-1
	393.79 ± 1.44 A,b	641.37	247.58 ± 1.15 A,b	Soil sediments	SS-2
	337.45 ± 1.42 E,b	576.17	238.72 ± 1.21 B,b	Soil sediments	SS-3
	352.37 ± 1.16 C,b	518.86	166.49 ± 1.19 E,b	Soil sediments	SS-4
	348.13 ± 1.40 D,b	537.89	189.76 ± 1.41 D,b	Soil sediments	SS-5
Pyrophosphate Group	73.75 ± 1.19 A,a	223.71	149.96 ± 1.22 A,a	Beach sediments	BS-1
	70.29 ± 1.04 A,a	210.62	140.33 ± 1.04 C,a	Beach sediments	BS-2
	77.45 ± 1.24 A,a	222.96	145.51 ± 1.14 B,a	Beach sediments	BS-3
	72.59 ± 1.07 A,a	206.36	133.77 ± 1.09 D,a	Beach sediments	BS-4
	76.63 ± 1.16 A,a	215.48	138.85 ± 1.05 C,a	Beach sediments	BS-5
UPW Group	40.75 ± 1.22 A,b	88.04	47.29 ± 1.08 B,b	Beach sediments	BS-1
	38.84 ± 1.34 A,b	85.37	46.53 ± 1.12 B,b	Beach sediments	BS-2
	39.37 ± 1.12 A,b	80.15	40.78 ± 1.08 C,b	Beach sediments	BS-3
	40.43 ± 1.11 A,b	90.89	50.46 ± 1.17 A,b	Beach sediments	BS-4
	37.75 ± 1.35 A,b	83.07	45.32 ± 1.08 B,b	Beach sediments	BS-5
UPW then Pyrophosphate Group	23.34 ± 1.34 B,c	123.79	100.45 ± 1.06 A,b	Beach sediments	BS-1
	33.73 ± 1.12 A,c	132.40	98.67 ± 1.12 A,b	Beach sediments	BS-2
	37.14 ± 1.08 A,b	138.76	101.62 ± 1.21 A,b	Beach sediments	BS-3
	34.54 ± 1.12 A,c	122.12	87.58 ± 1.15 B,b	Beach sediments	BS-4
	40.19 ± 1.03 A,b	135.82	95.63 ± 1.11 A,b	Beach sediments	BS-5

Values expressed as mean ± standard deviation obtained from 3 measurements. Capital letters indicate significant differences related to different sources in a certain group, while lowercase letters indicate significant differences related to different groups with the same sample type for a certain kind of source. Note: SS-1, SS-2, SS-3, SS-4 and SS-5 represented Jiangdong village, Jiangdongcun farm, Yangli farm, Xianmeng Jiangdong vineyard and Chunlai Li ecological farm, respectively. BS-1, BS-2, BS-3, BS-4 and BS-5 represented Jinshan city beach, Jinshan district coastal park, Parrot Island ecological wetland, Jinshanzui fishing village and Jinshan water base, respectively.

**Table 4:** TOC and IC concentrations in soil and beach sediments under different pretreatment.

Pretreatment Method	Mean of TOC (mg/L)	TC (mg/L)	Mean of IC (mg/L)	Microplastics	Sample Source
Pyrophosphate Group	252.34 ± 0.82 A,a	402.13	149.79 ± 1.01 A,a	EPE	SS-1
	249.71 ± 0.79 A,a	388.29	138.58 ± 0.85 B,a	EPS	SS-2
	251.35 ± 0.89 A,a	361.90	110.55 ± 0.88 C,a	FPUF	SS-3
	180.64 ± 1.01 B,a	251.36	70.72 ± 0.82 D,a	RPUF	SS-4
	80.27 ± 0.86 C,a	61.11	40.84 ± 0.83 E,a	LDPE	SS-5
UPW Group	151.00 ± 0.88 A,b	197.34	46.34 ± 0.85 A,b	EPE	SS-1
	158.70 ± 1.03 B,b	217.51	58.81 ± 1.23 B,b	EPS	SS-2
	141.95 ± 1.24 C,b	194.69	52.74 ± 1.36 C,b	FPUF	SS-3
	102.30 ± 1.05 D,b	142.03	39.73 ± 0.86 D,b	RPUF	SS-4
	72.90 ± 1.36 E,b	100.03	27.13 ± 0.85 E,b	LDPE	SS-5
UPW then Pyrophosphate Group	100.56 ± 1.44 A,c	203.23	102.67 ± 0.90 A,c	EPE	SS-1
	98.34 ± 0.98 A,c	185.69	87.35 ± 0.90 B,c	EPS	SS-2
	103.52 ± 0.86 B,c	163.84	60.63 ± 0.85 C,c	FPUF	SS-3
	81.17 ± 0.83 C,c	114.63	33.46 ± 0.82 D,c	RPUF	SS-4
	13.63 ± 0.88 D,c	29.05	15.42 ± 0.91 E,c	LDPE	SS-5
Pyrophosphate Group	65.30 ± 0.84 A,a	217.30	152.00 ± 0.88 A,a	EPE	BS-1
	67.65 ± 0.87 B,a	208.95	141.30 ± 0.89 B,a	EPS	BS-2
	70.85 ± 0.84 C,a	216.90	146.05 ± 0.97 C,a	FPUF	BS-3
	39.55 ± 0.70 D,a	141.30	101.75 ± 0.82 D,a	RPUF	BS-4
	19.20 ± 0.89 E,a	73.45	54.25 ± 1.13 E,a	LDPE	BS-5
UPW Group	37.80 ± 1.86 A,b	85.68	47.88 ± 0.95 A,b	EPE	BS-1
	35.90 ± 0.91 A,b	87.94	52.04 ± 1.31 B,b	EPS	BS-2
	43.85 ± 0.94 B,b	98.76	54.91 ± 0.88 C,b	FPUF	BS-3
	26.80 ± 1.13 C,b	68.75	41.95 ± 0.89 D,b	RPUF	BS-4
	12.65 ± 1.31 D,b	32.40	19.75 ± 0.90 E,b	LDPE	BS-5
UPW then Pyrophosphate Group	30.13 ± 0.90 A,c	137.48	107.35 ± 0.82 B,c	EPE	BS-1
	31.98 ± 0.59 A,c	128.21	96.23 ± 0.89 A,c	EPS	BS-2
	28.39 ± 0.57 A,c	123.12	94.73 ± 0.82 A,c	FPUF	BS-3
	14.46 ± 0.75 B,c	76.04	61.58 ± 0.88 C,c	RPUF	BS-4
	8.15 ± 0.71 C,c	45.02	36.87 ± 0.86 D,c	LDPE	BS-5

Values expressed as mean ± standard deviation obtained from 3 measurements. Capital letters indicate significant differences related to different sources in a certain pretreatment group, while lowercase letters indicate significant differences related to different pretreatment groups for a certain kind of source.

**Table 5:** Contents of DOM in different environmental media.

Environmental Medium	Sample Source	Mean of TOC (mg/L)	TC (mg/L)	Mean of IC (mg/L)	Microplastics
MPs in river water	River-1	4.28 ± 0.16 A,a	27.25	22.97 ± 0.10 A,a	EPE-MPs
	River-2	4.14 ± 0.16 A,a	24.71	20.57 ± 0.10 B,a	EPS-MPs
	River-3	3.93 ± 0.10 A,a	22.57	18.64 ± 0.19 C,a	FPUF-MPs
	River-4	2.17 ± 0.16 B,a	18.81	16.64 ± 0.18 D,a	RPUF-MPs
	River-5	1.12 ± 0.16 C,a	8.63	7.51 ± 0.19 E,a	LDPE-MPs
MPs in Qiantang river	QTR-1	3.88 ± 0.07 A,a	26.84	22.96 ± 0.18 A,a	EPE-MPs
	QTR-2	3.73 ± 0.11 A,b	24.57	21.84 ± 0.19 B,b	EPS-MPs
	QTR-3	3.65 ± 0.10 A,a	25.03	21.38 ± 0.18 C,b	FPUF-MPs
	QTR-4	1.34 ± 0.09 B,b	14.74	13.40 ± 0.11 D,b	RPUF-MPs
	QTR-5	1.06 ± 0.08 C,a	7.34	6.28 ± 0.16 E,b	LDPE-MPs
MPs in seawater	ECS-1	4.12 ± 0.09 A,a	19.29	15.17 ± 0.12 A,b	EPE-MPs
	ECS-2	4.03 ± 0.08 A,a	18.71	14.68 ± 0.12 B,c	EPS-MPs
	ECS-3	3.91 ± 0.10 A,a	17.48	13.57 ± 0.11 C,c	FPUF-MPs
	ECS-4	2.19 ± 0.10 B,a	10.77	8.58 ± 0.19 D,c	RPUF-MPs
	ECS-5	0.81 ± 0.09 C,a	5.16	4.35 ± 0.20 E,c	LDPE-MPs
MPs in soil sediments	SS-1	248.73 ± 0.11 A,b	396.05	147.32 ± 0.18 A,c	EPE-MPs
	SS-2	250.51 ± 0.08 B,c	391.07	140.56 ± 0.30 B,d	EPS-MPs
	SS-3	247.36 ± 0.18 C,b	357.11	109.75 ± 0.32 C,d	FPUF-MPs
	SS-4	182.49 ± 0.28 D,c	241.02	68.53 ± 0.24 D,d	RPUF-MPs
	SS-5	77.73 ± 0.57 E,b	119.52	41.79 ± 0.21 E,d	LDPE-MPs
MPs in beach sediments	BS-1	61.46 ± 0.18 A,c	211.25	149.79 ± 0.19 A,d	EPE-MPs
	BS-2	62.37 ± 0.11 B,d	204.93	142.56 ± 0.18 B,e	EPS-MPs
	BS-3	67.75 ± 0.10 C,c	208.07	140.32 ± 0.20 C,e	FPUF-MPs
	BS-4	38.99 ± 0.18 D,d	138.86	99.87 ± 0.19 D,e	RPUF-MPs
	BS-5	17.98 ± 0.18 E,c	68.35	50.37 ± 0.22 E,e	LDPE-MPs

Values expressed as mean ± standard deviation obtained from 3 measurements. Capital letters indicate significant differences related to different MPs in a certain Environmental medium group. Note: The meanings of a, b, c, d and e were the same as those in Table 1.

**Table 6:** Peak molecular weights and retention times of DOM adsorbed on five microplastics in different environments.

Media	MPs	Peak 1		Peak 2		Peak 3		Peak 4		Peak 5		Peak 6		Peak 7	
		Retention Time (min)	*M_w_* (Da)	Retention Time (min)	*M_w_* (Da)	Retention Time (min)	*M_w_* (Da)	Retention Time (min)	*M_w_* (Da)	Retention Time (min)	*M_w_* (Da)	Retention Time (min)	*M_w_* (Da)	Retention Time (min)	*M_w_* (Da)
River	EPE	8.556	5382	8.850	4552	9.268	3960	9.924	2774	10.756	1171	11.382	408	/	/
	EPS	8.562	5325	8.856	4536	9.270	3938	9.925	2756	10.753	1227	11.381	412	/	/
	FPUF	8.553	5440	8.850	4538	9.270	3960	9.926	2750	10.750	1267	11.383	401	/	/
	RPUF	8.525	5489	8.858	4522	9.262	3986	9.921	2788	10.754	1220	11.383	401	/	/
	LDPE	8.583	5288	8.856	4527	9.275	3910	9.925	2757	10.755	1217	11.381	411	/	/
QTR	EPE	8.542	5327	8.864	4518	9.290	3905	10.007	2628	10.864	1091	11.738	215	/	/
	EPS	8.556	5402	8.852	4540	9.282	3890	9.940	2698	10.859	1108	11.380	377	/	/
	FPUF	8.542	5360	8.848	4557	9.274	3970	9.924	2760	11.098	1162	11.375	495	/	/
	RPUF	8.546	5350	8.850	4530	9.281	3867	9.948	2630	10.749	1371	11.105	840	/	/
	LDPE	8.550	5370	8.851	4528	9.275	3972	9.938	2706	10.801	1312	11.100	805	/	/
ECS	EPE	8.589	5215	8.888	4497	9.303	3914	10.090	2517	10.940	1123	/	/	/	/
	EPS	8.618	5228	8.880	4498	9.301	3910	10.091	2514	10.875	1138	/	/	/	/
	FPUF	8.600	5210	8.823	4501	9.310	3902	10.106	2498	10.939	1165	/	/	/	/
	RPUF	8.601	5216	8.913	4465	9.302	3908	10.092	2512	10.827	1160	/	/	/	/
	LDPE	8.594	5203	8.766	4540	9.320	3888	10.080	2530	10.868	1140	/	/	/	/
SS	EPE	8.362	6190	8.672	4780	9.101	4148	9.749	3051	11.182	683	11.450	435	/	/
	EPS	8.045	7302	8.244	5360	8.545	4933	8.974	4305	9.603	3342	11.483	408	/	/
	FPUF	8.535	5215	9.015	4313	9.642	3299	10.698	1731	11.441	440	11.548	368	/	/
	RPUF	8.557	5179	8.856	4520	9.273	3940	9.974	2756	10.870	1146	11.550	362	/	/
	LDPE	8.534	5225	8.943	4466	9.728	3215	10.662	1701	10.672	1671	11.280	620	/	/
BS	EPE	8.368	6178	8.671	4790	9.099	4153	9.750	3047	11.185	676	/	/	/	/
	EPS	8.546	5450	8.860	4548	9.280	3970	9.944	2750	11.101	1028	11.393	481	/	/
	FPUF	8.510	5445	9.005	4358	9.638	3325	10.690	1750	11.442	438	/	/	/	/
	RPUF	8.553	5512	8.860	4510	9.274	3989	9.970	2757	10.862	1163	11.551	360	/	/
	LDPE	8.531	5234	8.941	4481	9.723	3364	10.665	1697	11.283	618	11.501	356	/	/

## Data Availability

We can provide and share experimental data in order to enable other authors to achieve best practices in archiving research data.
